# Editorial: Advancing precision therapies in glioblastoma

**DOI:** 10.3389/fonc.2025.1681786

**Published:** 2025-09-25

**Authors:** Theophilos Tzaridis, Marike Broekman, Konstantinos Gousias

**Affiliations:** ^1^ Cancer Genome and Epigenetics Program, NCI-Designated Cancer Center, Sanford Burnham, Prebys Medical Discovery Institute, La Jolla, CA, United States; ^2^ Department of Neurosurgery, Haaglanden Medical Center, The Hague, Netherlands; ^3^ Department of Neurosurgery, Athens Medical Center, Athens, Greece; ^4^ University of Münster Medical School, Münster, Germany; ^5^ European University of Cyprus Medical School, Nicosia, Cyprus

**Keywords:** glioblastoma, therapy, blood-brain barrier, targeted therapy, drug delivery, biomarkers

Glioblastoma (GB) is a devastating brain tumour with an urgent need for novel therapeutic strategies. This collection of reviews, case reports and basic research includes ten articles and covers a broad range of advances to treating GB, from solidifying our understanding of its biology to developing ways to overcome the blood-brain barrier (BBB) and targeted therapy.

The pillar of GB therapy remains tumour resection. Determining the resection margin for these tumours poses a major challenge. Koga et al. introduce a novel white matter tractography technique and a survey on its efficacy among neurosurgeons to allow for individualised corrections based on the underlying pathology. This novel method led to accurate assessments of lesion margins, edematous zones and a highly efficient, individualised preoperative planning.

The infiltrative nature and frequent occurrence of GB in eloquent brain regions render precise intraoperative mapping and monitoring (IOMM) techniques imperative. Staub-Bartlet et al. provide an overview of different IOMM techniques and demonstrate that their individualized use allows for maximal safe resections.

Further treatment modalities are irradiation and chemotherapy with alkylating agents. Recently, targeted therapy based on molecular alterations of tumours has emerged, yet failed to achieve durable successes for GB patients. An example of failed targeted therapies are receptor tyrosine kinase inhibitors, either targeting single tyrosine kinases (such as EGFR) or multi-kinase inhibitors (such as Imatinib or dovitinib). Nevertheless, Shahab et al. show a remarkable response of a patient with an infant-type bihemispheric glioma harbouring an ATIC-ALK fusion to ALK inhibitor lorlatinib indicating that molecularly-guided use of some of these compounds could potentially be beneficial for a subset of patients. Future studies are warranted to identify these patient subpopulations and understand mechanisms of resistance.

Immunotherapy has revolutionised treatment of patients with solid tumours, yet similar to targeted therapy has failed to do so for GB. One exciting immunotherapeutic approach is the adoptive cell treatment and the use of chimeric antigen receptor (CAR) NK cells. Xiong et al. discuss CAR-NKs in GB and mention advantages over CAR-Ts, such as reduced toxicity or their MHC-I independent mode of function, but also drawbacks, such as limited expansion efficacy *in vitro* and short survival time of the NK cells *in vivo*. The authors give an overview over seminal preclinical studies and clinical trials with the leading example of a Phase I trial investigating the role of CAR-NKs against HER2 in the treatment of refractory GB.

Therapy failure in GB is intricately linked to the unique challenges posed by the BBB, which mitigates the effects of systemic delivery. Pinkiewicz et al. discuss the intra-arterial cerebral infusion of drugs to overcome this challenge. Despite earlier encouraging data on safety and enhanced toxicity profile of this treatment, no data have yet demonstrated improved efficacy as compared to conventional intravenous administration. Future studies could explore the super-selective delivery options, but also investigate novel exciting methods of BBB disruption, such as focused ultrasound or convection-enhanced delivery (CED).

A novel approach to both overcome the BBB and target GB cells is proposed by Campelo et al., the high-frequency irreversible electroporation (H-FIRE). H-FIRE increases the membrane potential leading to defects in the cell membrane, targets the BBB and potentially induces immunogenicity. The authors show that H-FIRE improved survival in rat glioma models and that it led to recruitment of CD8+ T cells, as well as upregulation of proinflammatory cytokines. An intriguing future combination could include H-FIRE and CED to maximise intratumoural drug levels by absorbing drug levels that would otherwise have been eliminated.

Another strategy using this double-pronged approach is presented by Shipley et al., who generated a Shigella strain that preferentially infects GB cells at a much higher rate as compared to normal astrocytes or neurons. This strain targets tumour cells either via direct killing or modification of the microenvironment. A further advantage over existing approaches, such as viral therapy, is the retained sensitivity of Shigella to classic antibiotics, yet its safety profile should be carefully assessed *in vivo* and its efficacy compared to current strategies.

A prerequisite for advancing GB therapies is our understanding of GB biology and the development of reliable *in vitro* models recapitulating the complexity of GB cells and their microenvironment. Xie et al. discuss different platforms integrating these components into a chip. The microfluidic polymeric chip has enabled the co-culture of different cell types, such as GB and endothelial cells thereby mimicking tumour vasculature. More recently, 3D bioprinting has been capable of integrating tumour cells with immune cells and components of the extracellular matrix and excitingly does so using patient-derived GB cells. One intriguing example is the creation of a GBM-on-a-chip to study resistance of GB to immune checkpoint blockade by including components of the immunosuppressive tumour microenvironment.

Jin et al. performed a mostly *in silico* study focussing on the glycosylation of proteins in GB tumours and saw that expression of regulators of glycosylation correlates with distinct clinicopathological features of GB and response to certain therapies. For a subset of tumours harbouring glycosylation changes, they identified Clorafabin and YM-155 as potential drug candidates. If confirmed in different patient cohorts, this platform could be an exciting additional tool for clinical decision making. Importantly, this study draws attention to the post-translational modifications of GB cells, which are frequently overlooked.

Finally, integrating tumour biology, therapeutic delivery and biomarkers, Lunavat et al. from the Breakefield team discuss the different roles of extracellular vesicles (EVs) in GB. EVs from tumour cells can infect healthy bystander cells, remodel tumour vasculature and even suppress an antitumoural immune response. Conversely, they can also travel to lymph nodes, present neoantigens and stimulate cytotoxic T cells. Increasing attention is being drawn to the use of nanoparticles as deliverers of chemotherapy, but also novel treatments such as antisense oligonucleotides. Finally, the authors mention the emerging role of EVs as biomarkers for GB patients making them protagonists of modern liquid biopsy strategies.

This Research Topic offers a variety of novel ideas to advance GB therapy. It underscores the need for an integrative approach addressing challenges at the surgical level, improving our understanding of tumour biology and thereby molecular vulnerabilities, achieving therapeutic delivery by disrupting the BBB and using novel modalities such as immunotherapy. Only with this multimodal perspective will we be able to overcome past failures and ultimately improve survival of patients with this devastating disease.

